# Naringin derivatives as glucosamine-6-phosphate synthase inhibitors based preservatives and their biological evaluation

**DOI:** 10.1038/s41598-020-77511-2

**Published:** 2020-11-24

**Authors:** Amit Lather, Sunil Sharma, Anurag Khatkar

**Affiliations:** 1 Laboratory for Preservation Technology and Enzyme Inhibition Studies, Faculty of Pharmaceutical Sciences, Maharshi Dayanand University, Rohtak, Haryana India; 2 Department of Pharmaceutical Sciences, G.J.U.S.&T., Hisar, India

**Keywords:** Biochemistry, Biotechnology, Computational biology and bioinformatics, Microbiology

## Abstract

Glucosamine-6-Phosphate synthase enzyme has been targeted for development of better and safe preservative due to its role in microbial cell wall synthesis. In recent year’s demand of preservatives for the food, cosmetics and pharmaceuticals have increased. Although, the available synthetic preservatives have associated unwanted adverse effects, soa chain of naringin derivatives were schemed synthesized and judged for antioxidant, antimicrobial, preservative efficacy, stability study and topical evaluation. Molecular docking resulted with excellent dock score and binding energy for compound **7**, compound **6** and compound **1** as compared to standard drugs. Resultant data of antimicrobial activity revealed compound **7**as most potent antimicrobial compound for *P. mirabilis, P. aeruginosa, S. aureus*, *E. coli, C. albicans,* and *A. niger*, respectively*,* as compared to the standard drugs. The preservative efficacy test of compound **7** in White Lotion USP showed the log cfu/mL value within prescribed limit of USP standard. Compound **7** stabilize the White lotion USP from microbial growth for a period of six months under accelerated storage condition. Compound **7** was further evaluated for toxicity by using the Draize test in rabbits and showed no sign of eye and skin irritation. The outcome demonstrated that synthesized naringin compounds showed glorious antioxidant, antimicrobial, preservative efficacy, stable and safe as compared to standards.

## Introduction

Foods, cosmetics, and pharmaceutical products containing aqueous base have been reported with a higher risk of microbial growth and undesirable chemical changes. Preservatives like benzoic acid, methylparaben, ethylparaben, sodium benzoate, chlorobutanol, benzyl alcohol, phenyl ethyl alcohol, benzalkonium chloride, etc. have been added to food, pharmaceuticals, biological and other products to improve their shelf life^1–4^.

Most of the commercially used preservatives are of synthetic origin and they are associated with harmful side effects viz*.* cancer, Alzheimer disease, Parkinson disease, type-II diabetes, headache, nausea, weakness, asthma, neurological damage, irritation, allergies, etc.^5–12^. Hence, there is an urgent need for the search of better, safe and natural preservatives for food, cosmetics and pharmaceutical products. Hence, the various research groups have explored the medicinal plants for their specific antimicrobial and antioxidant along with the preservative efficacy. Among the natural compounds the ferulic acid, gallic acid, caffeic acid, p-coumaric acid, rosemerinic acid etc. have been explored for their preservative potential^13–15^.

Naringin, (7-[[2-*O*-(6-Deoxy-α-l-mannopyranosyl)-β-d-glucopyranosyl]oxy]-2,3-dihydro-5-hydroxy-2-(4-hydroxyphenyl)-4*H*-1-benzopyran-4-one) obtained from the grapefruit, lemon, orange juice, pummel, vegetables, *Ziziphus spina* and *Leptospermum scoparium,* etc.^16–22^. It has been reported to have the potential in the treatment of various human diseases viz. inflammation, cardiovascular disease, antioxidant, diabetes, dyslipidemia, immune system disease, allergy, cancer, neurodegenerative disorder, osteoporosis and neurotrophic effect, etc.^23–34^. Further, the naringin has also been explored in various animal models for anxiety, Parkinson’s disease, sedation, convulsion, neuroprotection, etc.^35,36^. Naringin and its derivatives viz*.* naringenin, prunin and alkylprunin esters have also been reported to have potent antibacterial activity against pathogenic bacteria *L. monocytogenes*, *E. coli* and *S. aureus*^37,38^.

These pharmacological activities of naringin made it a potential candidate for the discovery of novel antimicrobial preservatives.G-6-P synthase is a complex enzyme involved in the formation of UDP-N-acetyl glucosamine and catalyzes the initial step in hexosamine biosynthesis. One of these catalyzed products, N-acetyl glucosamine, is an important part of the peptidoglycan layer of bacterial and fungal cell wall. G-6-P synthase enzyme has been involved in the synthesis of microbial cell wall has been targeted by many researchers including our team for the discovery of new antimicrobials^39–41^. Docking software’s are handy for the screening of thousands of molecules affinity towards a particular disease target and the availability of three-dimensional structure of enzyme G-6-Psynthase (pdb id 1moq) for docking study shall enable the researchers to work in dry lab to develop its inhibitors^42–45^. In the present work the in-silico studies for proposed naringin derivatives, their synthesis, evaluation for their antioxidant, antimicrobial, preservative efficacy, stability study, skin membrane permeation study as well as the in-vivo topical and ocular toxicity has been reported.

## Experimental

### Material and methods

All the analytical grade chemicals were used in the present study and purchased from Loba Chemie (Mumbai, India), Sigma Aldrich (Germany) and SRL (Mumbai, India). Microbiological media like nutrient agar, nutrient broth, and sabouraud dextrose agar and sabouraud dextrose broth were obtained from Hi-media laboratories (Mumbai, India). Standard antimicrobials like streptomycin, ciprofloxacin, ampicillin and fluconazole were obtained from Belco Pharma (Bahadurgarh, India). The standard microbial strains *S. aureus* MTCC 3160, *P. aeruginosa* MTCC 1934, *E. coli* MTCC 45, *C. albicans* MTCC 183 and *A. niger* MTCC 282 in lyophilized form were purchased from MTCC, Chandigarh, India. Melting point of naringin derivatives were recorded by Sonar melting point apparatus. Infrared (IR) spectra were recorded on Perkin Elmer FTIR spectrophotometer.^1^H NMR and ^13^C NMR spectra were recorded on Bruker Avance II 400 NMR spectrometer. Mass spectra were recorded on Waters Micromass Q-ToF Micro instrument. Elemental analysis was checked on Perkin-Elmer 2400 CHN analyzer. Institutional Animal Ethical Committee of M.D. University, Rohtak, India has approved the experimental protocol for use of rabbits vide letter no. 1767/GO/Re/S/14/CPCSEA, dated- 31/08/2017. Young adult albino rabbits were procured from Lala Lajpat Rai University of Veterinary and Animal Sciences, (Hisar, India). All experiments were performed in accordance with relevant guidelines and regulations.

### In silico molecular docking studies

The Schrodinger, Inc. software platform Maestro 10 was used for docking calculations. Laboratory for Preservation Technology and Enzyme Inhibition Studies, Department of Pharmaceutical Sciences, M.D. University, Rohtak, India was used for computational work. The receptor-grid files were generated by grid-receptor generation program (Glide). A conjugate gradient minimization protocol was used in all calculations^46^.

The energy differences were calculated using the equation:

$$\Delta E = E_{{{\text{complex}}}} - E_{{{\text{ligand}}}} - E_{{{\text{protein}}}}$$

### Protein preparation

Pdb id 1moq (with minimum resolution 1.57 Å) was selected and downloaded from Protein Data Bank. Protein structure was prepared with the protein preparation wizard Prepwiz. During the protein preparation all the water molecules except those coordinated to metals as well between ligand and protein were removed. The energy restrained structure of targeted protein was constructed by using OPLS-2005 force field.

### Ligand preparation

The three-dimensional structural of naringin derivatives were constructed by using the Chemdraw ultra 8 and were further preceded for energy minimization by using the LigPrep tool to gain the appropriate conformation through the addition or removal of hydrogen bonds. The partial charges were computed according to the OPLS-2005 force field at biological pH.

### ADME studies

In silico prediction for ADME properties of the synthesized compounds were calculated by quick prop from Schrodinger. Various ADME parameters such as Log P, number of rotatable bonds, Log BB, number of hydrogen acceptor and donor atoms were calculated. Lipinski’s rule of five was used for the prediction of drug-likeness properties of synthesized naringin derivatives.

### General procedure for the synthesis of naringin derivatives

**Scheme 1:**
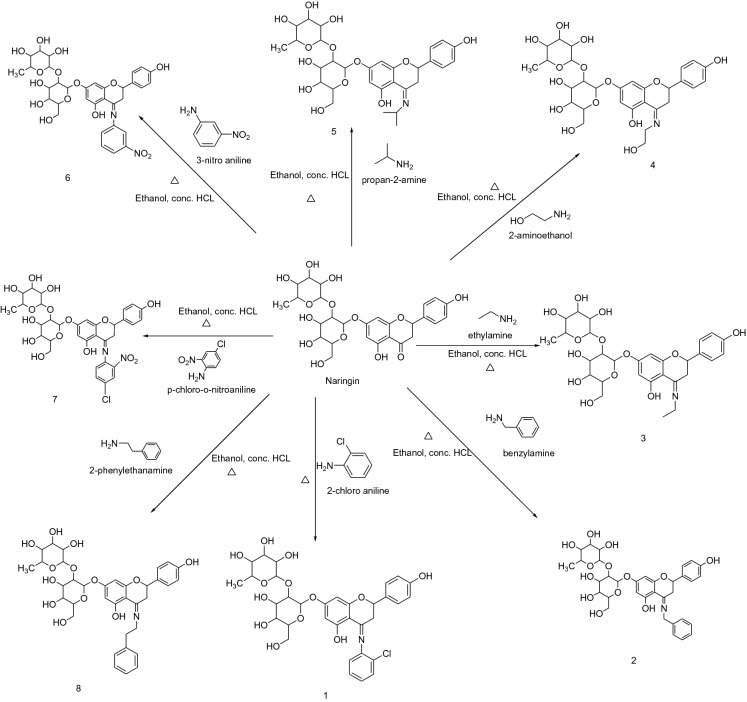
Synthetic route for naringin derivatives.

The naringin derivatives were synthesized as per the procedure of Yang et al. and Saini et al. with slide modifications and are outlined in Scheme 1^47,48^. Substituted aniline (0.01 mol) was taken in a round bottom flask and concentrated hydrochloric acid drop wise was added. Equimolar concentration of naringin (0.01 mol) was dissolved in ethanol (50 mL) in equimolar concentration and was refluxed. Completion of the reaction was confirmed by single spot TLC. After the completion of reaction the concentrated reaction mixture was concentrated and the formed precipitated were filtered off desiccated. The crude products were recrystallized using alcohol yielded compound **1**–**8**. The confirmation of the final compounds was made by physicochemical and spectral methods like FTIR, ^1^H NMR and ^13^C NMR spectra, Mass spectroscopy and elemental analysis.

### Spectral data

#### Compound **1**: 2-(2-(4-(2-chlorophenylimino)-5-hydroxy-2-(4-hydroxyphenyl)-3,4-dihydro-2*H*-chromen-7-yloxy)-4,5-dihydroxy-6-(hydroxymethyl)-tetrahydro-2*H*-pyran-3-yloxy)-6-methyl-tetrahydro-2*H*-pyran-3,4,5-triol

M.p.: 60–62 °C; TLC (Chloroform:ethanol: 5:1 v/v): R_f _= 0.67; Yield = 38.97%; M.Wt. = 690.11; IR (KBr pellets) cm^−1^: 746 (–Cl– str), 1077 (–C–O–C), 1202 (–C–C–), 1602 (–C=C–), 1638 (–C=N–), 2927 (–C–H–), 3381 (–OH–); ^1^H NMR (400 MHz, DMSO-*d*_6_) δ = 1H NMR (400 MHz, DMSO-d6) δ 8.86 (s, 1H), 8.04 (s, 1H), 7.24–7.17 (m, 3H), 7.15 (dd, J = 7.5, 1.5 Hz, 1H), 6.95 (dd, J = 7.5, 1.5 Hz, 1H), 6.83–6.75 (m, 2H), 6.66 (td, J = 7.5, 1.5 Hz, 1H), 6.27 (dd, J = 14.2, 1.6 Hz, 2H), 5.28 (tt, J = 6.9, 0.7 Hz, 1H), 5.15 (d, J = 6.9 Hz, 1H), 5.02–4.88 (m, 3H), 4.79–4.69 (m, 2H), 4.65 (d, J = 8.6 Hz, 1H), 4.43 (d, J = 8.1 Hz, 1H), 3.90–3.79 (m, 2H), 3.79–3.73 (m, 1H), 3.73–3.65 (m, 2H), 3.65–3.57 (m, 2H), 3.55–3.40 (m, 3H), 3.30–3.11 (m, 3H), 2.63 (dt, J = 12.4, 7.0 Hz, 1H), 2.52 (dt, J = 12.4, 7.0 Hz, 1H), 1.08 (dd, J = 6.7, 1.5 Hz, 3H); ^13^C NMR (400 MHz, CDCL_3_) δ = 161.86, 161.84, 158.72, 157.99, 141.99, 133.04, 130.31, 128.53, 127.05, 125.56, 124.66, 120.12, 116.25, 104.88, 103.35, 101.14, 97.13, 96.49, 78.89, 77.04, 75.71, 75.46, 72.64, 71.58, 71.28, 71.10, 70.97, 62.67, 44.27, 36.22, 16.17.; MS ES + (ToF): m/z 690.9 [M^+^ + 2]; CHNS: Calc (C_33_H_36_ClNO_13_): C, 57.43; H, 5.26; Cl, 5.14; N, 2.03; O, 30.14; Found C, 57.42; H, 5.27; Cl, 5.13; N, 2.02; O, 30.16.

#### Compound **2**: 2-(2-(4-(benzylimino)-5-hydroxy-2-(4-hydroxyphenyl)-3,4-dihydro-2*H*-Chromen-7-yloxy)-4,5-dihydroxy-6-(hydroxymethyl)-tetrahydro-2*H*-pyran-3-yloxy)-6-methyl-tetrahydro-2*H*-pyran-3,4,5-triol

M.p.: 65–67 °C; TLC (Chloroform:Methanol: 5:1 v/v): R_f_ = 0.68; Yield = 30.90%; M.Wt. = 669.67; IR (KBr pellets) cm^−1^: 1073 (–C–O–C), 1171 (–C–C–), 1602 (–C = C–), 1642 (–C=N–), 3029 (–C–H–), 3359 (–OH–); ^1^H NMR (400 MHz, CDCL_3_) δ = 1H NMR (400 MHz, DMSO-d6) δ 8.86 (s, 1H), 8.04 (s, 1H), 7.38–7.29 (m, 2H), 7.32–7.23 (m, 1H), 7.27–7.14 (m, 4H), 6.83–6.75 (m, 2H), 6.27 (dd, J = 14.2, 1.6 Hz, 2H), 5.26 (tt, J = 7.0, 0.7 Hz, 1H), 5.15 (d, J = 6.9 Hz, 1H), 4.99–4.88 (m, 2H), 4.76 (d, J = 8.8 Hz, 1H), 4.65 (d, J = 8.6 Hz, 1H), 4.43 (d, J = 8.1 Hz, 1H), 4.32 (dt, J = 8.5, 7.0 Hz, 1H), 3.94–3.79 (m, 4H), 3.83–3.68 (m, 1H), 3.73–3.62 (m, 2H), 3.61 (t, J = 7.0 Hz, 1H), 3.55–3.40 (m, 3H), 3.30–3.11 (m, 3H), 3.08 (q, J = 8.4 Hz, 1H), 2.55 (dt, J = 12.3, 7.0 Hz, 1H), 2.44 (dt, J = 12.3, 7.0 Hz, 1H), 1.08 (dd, J = 6.7, 1.5 Hz, 3H); ^13^C NMR (400 MHz, CDCL_3_) δ = 161.59, 161.38, 158.52, 157.99, 139.02, 133.04, 128.53, 128.15, 128.11, 127.42, 116.25, 105.63, 103.35, 100.84, 97.13, 96.63, 78.89, 77.04, 75.71, 75.46, 72.64, 71.58, 71.28, 71.10, 70.97, 62.67, 53.14, 51.29, 37.33, 16.17; MS ES + (ToF): m/z 669.24 [M^+^ + 2]; CHNS: Calc (C_34_H_39_NO_13_): C, 60.98; H, 5.87; N, 2.09; O, 31.06; Found C, 60.95; H, 5.90; N, 2.08; O, 31.07.

#### Compound **3**: 2-(2-(4-(ethylimino)-5-hydroxy-2-(4-hydroxyphenyl)-3,4-dihydro-2*H*-chromen-7-yloxy)-4,5-dihydroxy-6-(hydroxymethyl)-tetrahydro-2*H*-pyran-3-yloxy)-6-methyl-tetrahydro-2*H*-pyran-3,4,5-triol

M.p.: 180–182 °C; TLC (Chloroform:Methanol: 5:1 v/v): R_f_ = 0.61; Yield = 52.78%; M.Wt. = 607.23; IR (KBr pellets) cm^−1^: 1063 (–C–O–C), 1174 (–C–C–), 1553 (–C=C–), 1687 (–C=N–), 2929 (–C–H–), 3387 (–OH–); ^1^H NMR (400 MHz, CDCL_3_) δ = 1H NMR (400 MHz, DMSO-d6) δ 8.86 (s, 1H), 8.04 (s, 1H), 7.24–7.17 (m, 2H), 6.83–6.75 (m, 2H), 6.27 (dd, J = 14.2, 1.6 Hz, 2H), 5.26 (tt, J = 7.1, 0.7 Hz, 1H), 5.15 (d, J = 6.9 Hz, 1H), 4.99–4.88 (m, 2H), 4.76 (d, J = 8.8 Hz, 1H), 4.65 (d, J = 8.6 Hz, 1H), 4.43 (d, J = 8.1 Hz, 1H), 4.29 (dt, J = 8.2, 7.0 Hz, 1H), 3.90–3.79 (m, 2H), 3.79–3.68 (m, 1H), 3.73–3.62 (m, 2H), 3.61 (t, J = 7.0 Hz, 1H), 3.55–3.40 (m, 3H), 3.30–3.11 (m, 3H), 2.91–2.70 (m, 3H), 2.52 (dt, J = 12.3, 7.0 Hz, 1H), 2.41 (dt, J = 12.4, 7.0 Hz, 1H), 1.25–1.15 (m, 3H), 1.08 (dd, J = 6.7, 1.5 Hz, 3H); ^13^C NMR (400 MHz, CDCL_3_) δ = 161.59, 161.38, 158.52, 157.99, 133.04, 128.53, 116.25, 105.63, 103.35, 100.84, 97.13, 96.63, 78.89, 77.04, 75.71, 75.46, 72.64, 71.58, 71.28, 71.10, 70.97, 62.67, 52.23, 42.10, 37.33, 16.17, 15.13; MS ES + (ToF): m/z 607.23 [M^+^ + 2]; CHNS: Calc (C_29_H_37_NO_13_): C, 57.33; H, 6.14; N, 2.31; O, 34.23; Found C, 57.35; H, 6.12; N, 2.30; O, 34.22.

#### Compound **4**: (Z)-2-(4,5-dihydroxy-2-(5-hydroxy-4-(2-hydroxyethylimino)-2-(4-hydroxyphenyl)-3,4-dihydro-2*H*-chromen-7-yloxy)-6-(hydroxymethyl)-tetrahydro-2*H*-pyran-3-yloxy)-6-methyl-tetrahydro-2*H*-pyran-3,4,5-triol

M.p. = 130–132 °C; TLC (Chloroform:Methanol: 5:1 v/v):R_f_ = 0.65; Yield = 32.35%; M.Wt. = 623.6; IR (KBr pellets) cm^−1^: 1081 (–C–O–C), 1179 (–C–C–), 1628 (–C=C–), 1654 (–C=N–), 2929 (–C–H–), 3257 (–OH–); ^1^H NMR (400 MHz, CDCL_3_) δ = 8.86 (s, 1H), 8.04 (s, 1H), 7.50–7.37 (m, 4H), 7.32–7.23 (m, 1H), 7.27–7.17 (m, 2H), 6.83–6.75 (m, 2H), 6.27 (dd, J = 14.2, 1.6 Hz, 2H), 5.27 (tt, J = 6.9, 0.7 Hz, 1H), 5.15 (d, J = 6.9 Hz, 1H), 4.99–4.88 (m, 2H), 4.76 (d, J = 8.8 Hz, 1H), 4.65 (d, J = 8.6 Hz, 1H), 4.43 (d, J = 8.1 Hz, 1H), 4.34–4.21 (m, 2H), 3.90–3.79 (m, 2H), 3.79–3.68 (m, 1H), 3.73–3.62 (m, 2H), 3.61 (t, J = 7.0 Hz, 1H), 3.55–3.40 (m, 3H), 3.30–3.11 (m, 3H), 2.96 (dd, J = 9.1, 8.5 Hz, 1H), 2.55 (dt, J = 12.3, 7.0 Hz, 1H), 2.44 (dt, J = 12.3, 7.0 Hz, 1H), 1.51 (d, J = 6.9 Hz, 3H), 1.08 (dd, J = 6.7, 1.4 Hz, 3H); ^13^C NMR (400 MHz, CDCL_3_) δ = 158.62, 157.99, 145.53, 133.04, 128.53, 128.18, 126.65, 126.00, 116.25, 105.27, 103.35, 101.00, 97.13, 96.56, 78.89, 77.04, 75.71, 75.46, 72.64, 71.58, 71.28, 71.10, 70.97, 62.67, 55.92, 51.69, 37.84, 23.74, 16.17; MS ES + (ToF): m/z 623.6 [M^+^ + 2]; CHNS: Calc (C_29_H_37_NO_14_): C, 56.38; H, 6.36; N, 2.05; O, 35.21; Found C, 56.36; H, 6.38; N, 2.09; O, 35.84.

#### Compound **5**: (Z)-2-(4,5-dihydroxy-2-(5-hydroxy-2-(4-hydroxyphenyl)-4-(isopropylimino)-3,4-dihydro-2*H*-chromen-7-yloxy)-6-(hydroxymethyl)-tetrahydro-2*H*-pyran-3-yloxy)-6-methyl-tetrahydro-2*H*-pyran-3,4,5-triol

M.p. = 125–127 °C; TLC (Chloroform:Methanol: 5:1 v/v):R_f_ = 0.66; Yield = 23.22%; M.Wt. = 621.63; IR (KBr pellets) cm^−1^: 1054 (–C–O–C), 1156 (–C–C–), 1602 (–C=C–), 1627 (–C=N–), 2950 (–C–H–), 3414 (–OH–); ^1^H NMR (400 MHz, CDCL_3_) δ = 8.86 (s, 1H), 8.04 (s, 1H), 7.24–7.17 (m, 2H), 6.83–6.75 (m, 2H), 6.27 (dd, J = 14.2, 1.6 Hz, 2H), 5.27 (tt, J = 6.9, 0.7 Hz, 1H), 5.15 (d, J = 6.9 Hz, 1H), 4.99–4.88 (m, 2H), 4.76 (d, J = 8.8 Hz, 1H), 4.65 (d, J = 8.6 Hz, 1H), 4.43 (d, J = 8.0 Hz, 1H), 4.22 (dt, J = 9.5, 7.0 Hz, 1H), 3.90–3.78 (m, 2H), 3.81–3.67 (m, 1H), 3.71–3.61 (m, 2H), 3.60 (d, J = 6.9 Hz, 1H), 3.57–3.38 (m, 3H), 3.30–3.11 (m, 3H), 2.59–2.43 (m, 2H), 2.48–2.37 (m, 1H), 2.05 (dd, J = 11.2, 9.5 Hz, 1H), 1.53–1.31 (m, 4H), 1.08 (dd, J = 6.7, 1.5 Hz, 3H), 0.90 (t, J = 8.0 Hz, 6H); ^13^C NMR (400 MHz, CDCL_3_) δ = 161.71, 161.63, 158.62, 157.99, 133.04, 128.53, 116.25, 105.27, 103.35, 101.00, 97.13, 96.56, 78.89, 77.04, 75.71, 75.46, 72.64, 71.58, 71.28, 71.10, 70.97, 62.67, 61.08, 52.03, 37.84, 28.26, 16.17, 10.17; MS ES + (ToF): m/z 621.61 [M^+^ + 2]; CHNS: Calc (C_30_H_39_NO_13_): C, 57.96; H, 6.32; N, 2.25; O, 33.46; Found C, 57.95; H, 6.33; N, 2.25; O, 33.45.

#### Compound **6**: 2-(4,5-dihydroxy-2-(5-hydroxy-2-(4-hydroxyphenyl)-4-(3-nitrophenylimino)-3,4-dihydro-2*H*-chromen-7-yloxy)-6-(hydroxymethyl)-tetrahydro-2*H*-pyran-3-yloxy)-6-methyl-tetrahydro-2*H*-pyran-3,4,5-triol

M.p.: 140–142 °C; TLC (Chloroform:Methanol: 5:1 v/v): R_f_ = 0.65; Yield = 60.56%; M.Wt. = 700.64; IR (KBr pellets) cm^−1^: 990 (–C–O–C), 1082 (–C–C–), 1344 (–NO_2_), 1518 (–C=C–), 1625 (–C=N–), 2927 (–C–H–), 3324 (–OH–); ^1^H NMR (400 MHz,CDCL_3_) δ = 8.86 (s, 1H), 8.04 (s, 1H), 8.03–7.96 (m, 2H), 7.24–7.17 (m, 2H), 6.93–6.85 (m, 2H), 6.83–6.75 (m, 2H), 6.27 (dd, J = 14.2, 1.6 Hz, 2H), 6.14 (d, J = 10.7 Hz, 1H), 5.28 (tt, J = 6.9, 0.7 Hz, 1H), 5.15 (d, J = 6.9 Hz, 1H), 4.99–4.85 (m, 3H), 4.76 (d, J = 8.8 Hz, 1H), 4.65 (d, J = 8.6 Hz, 1H), 4.43 (d, J = 8.1 Hz, 1H), 3.90–3.79 (m, 2H), 3.74 (ddd, J = 12.4, 7.4, 6.8 Hz, 1H), 3.71–3.62 (m, 2H), 3.61 (t, J = 7.0 Hz, 1H), 3.55–3.40 (m, 3H), 3.30–3.11 (m, 3H), 2.62 (dt, J = 12.3, 7.0 Hz, 1H), 2.51 (dt, J = 12.4, 7.0 Hz, 1H), 1.08 (dd, J = 6.7, 1.4 Hz, 3H); ^13^C NMR (400 MHz, CDCL_3_) δ = 161.86, 161.84, 158.72, 157.99, 151.03, 141.99, 133.04, 128.53, 125.52, 116.81, 116.25, 104.88, 103.35, 101.14, 97.13, 96.49, 78.89, 77.04, 75.71, 75.46, 72.64, 71.58, 71.28, 71.10, 70.97, 62.67, 44.72, 36.22, 16.17; MS ES + (ToF): m/z 700.21 [M^+^ + 2]; CHNS: Calc (C_33_H_36_N_2_O_15_): C, 56.57; H, 5.18; N, 4.00; O, 34.25; Found C, 56.59; H, 5.16; N, 4.01; O, 34.23.

#### Compound **7**: 2-(2-(4-(4-chloro-2-nitrophenylimino)-5-hydroxy-2-(4-hydroxyphenyl)-3,4-dihydro-2*H*-chromen-7-yloxy)-4,5-dihydroxy-6-(hydroxymethyl)-tetrahydro-2*H*-pyran-3-yloxy)-6-methyl-tetrahydro-2*H*-pyran-3,4,5-triol

M.p. = 130–132 °C; TLC(Chloroform:Methanol: 5:1 v/v): R_f_ = 0.71; Yield = 75.55%; M.Wt. = 734.17; IR (KBr pellets) cm^−1^: 764 (–Cl– Str), 1090 (–C–O–C), 1250 (–C–C–), 1341 (–NO_2_), 1601 (–C=C–), 1639 (–C=N–), 2925 (–C–H–), 3474 (–OH–); ^1^H NMR (400 MHz, CDCL_3_) δ = 8.86 (s, 1H), 8.14 (d, J = 1.6 Hz, 1H), 8.07–7.99 (m, 2H), 7.24–7.17 (m, 2H), 6.90 (d, J = 7.4 Hz, 1H), 6.83–6.75 (m, 2H), 6.27 (dd, J = 14.2, 1.6 Hz, 2H), 5.78 (d, J = 10.5 Hz, 1H), 5.28 (tt, J = 6.9, 0.7 Hz, 1H), 5.15 (d, J = 6.9 Hz, 1H), 5.02–4.88 (m, 3H), 4.76 (d, J = 8.8 Hz, 1H), 4.65 (d, J = 8.6 Hz, 1H), 4.43 (d, J = 8.1 Hz, 1H), 3.90–3.79 (m, 2H), 3.74 (ddd, J = 12.4, 7.4, 6.8 Hz, 1H), 3.71–3.62 (m, 2H), 3.61 (t, J = 7.0 Hz, 1H), 3.55–3.40 (m, 3H), 3.30–3.11 (m, 3H), 2.63 (dt, J = 12.4, 7.0 Hz, 1H), 2.52 (dt, J = 12.4, 7.0 Hz, 1H), 1.08 (dd, J = 6.7, 1.4 Hz, 3H); ^13^C NMR (400 MHz, CDCL_3_) δ = 161.86, 161.84, 158.72, 157.99, 148.39, 140.87, 133.04, 128.53, 126.05, 125.57, 121.70, 119.23, 116.25, 104.88, 103.35, 101.14, 97.13, 96.49, 78.89, 77.04, 75.71, 75.46, 72.64, 71.58, 71.28, 71.10, 70.97, 62.67, 44.27, 36.22, 16.17; MS ES + (ToF): m/z 734.17 [M^+^ + 2]; CHNS: Calc (C_33_H_35_ClN_2_O_15_): C, 53.92; H, 4.80; Cl, 4.82; N, 3.81; O, 32.65; Found C, 53.91; H, 4.81; Cl, 4.80; N, 3.80; O, 32.66.

#### Compound **8**: 2-(2-(4-(benzylimino)-5-hydroxy-2-(4-hydroxyphenyl)-3,4-dihydro-2*H*-chromen-7-yloxy)-4,5-dihydroxy-6-(hydroxymethyl)-tetrahydro-2*H*-pyran-3-yloxy)-6-methyl-tetrahydro-2*H*-pyran-3,4,5-triol

M.p.: 128–130 °C; TLC (Chloroform:Methanol: 5:1 v/v); R_f_ = 0.64; Yield = 55.43%; M.Wt. = 683.7; IR (KBr pellets) cm^−1^: 1029 (–C–O–C), 1170 (–C–C–), 1602 (–C=C–), 1642 (–C=N–), 2875 (–C–H–), 3371 (–OH–); ^1^H NMR (400 MHz, CDCL_3_) δ = 8.86 (s, 1H), 8.04 (s, 1H), 7.50–7.37 (m, 4H), 7.32–7.23 (m, 1H), 7.27–7.17 (m, 2H), 6.83–6.75 (m, 2H), 6.27 (dd, J = 14.2, 1.6 Hz, 2H), 5.27 (tt, J = 6.9, 0.7 Hz, 1H), 5.15 (d, J = 6.9 Hz, 1H), 4.99–4.88 (m, 2H), 4.76 (d, J = 8.8 Hz, 1H), 4.65 (d, J = 8.6 Hz, 1H), 4.43 (d, J = 8.1 Hz, 1H), 4.34–4.21 (m, 2H), 3.90–3.79 (m, 2H), 3.79–3.68 (m, 1H), 3.73–3.62 (m, 2H), 3.61 (t, J = 7.0 Hz, 1H), 3.55–3.40 (m, 3H), 3.30–3.11 (m, 3H), 2.96 (dd, J = 9.1, 8.5 Hz, 1H), 2.55 (dt, J = 12.3, 7.0 Hz, 1H), 2.44 (dt, J = 12.3, 7.0 Hz, 1H), 1.51 (d, J = 6.9 Hz, 3H), 1.08 (dd, J = 6.7, 1.4 Hz, 3H); ^13^C NMR (400 MHz, CDCL_3_) δ = 158.62, 157.99, 145.53, 133.04, 128.53, 128.18, 126.65, 126.00, 116.25, 105.27, 103.35, 101.00, 97.13, 96.56, 78.89, 77.04, 75.71, 75.46, 72.64, 71.58, 71.28, 71.10, 70.97, 62.67, 55.92, 51.69, 37.84, 23.74, 16.17; MS ES + (ToF): m/z 683.26 [M^+^ + 2]; CHNS: Calc (C_35_H_41_NO_13_): C, 61.49; H, 6.04; N, 2.05; O, 30.42; Found C, 61.48; H, 6.02; N, 2.05; O, 30.40.

### Antioxidant activity

#### DPPH radical scavenging assay

Antioxidant activity of the synthesized was evaluated by photocolorimetric assay by using DPPH (2,2-diphenyl-1-pycrilhydrazil hydrate) free radical scavenging method. Briefly, 0.1 mM solution of DPPH was prepared in methyl alcohol and 1 mL of this solution was added in to 1 mL of sample or standard. Discolorations were measured at 517 nm after incubation for 30 min at 30 °C in the dark. Lesser absorbance of the reaction mixture indicates the higher free radical scavenging potential. The test was performed in triplicate and the % inhibition values of all the synthesized compounds were calculated by using the formula:

$$\% {\text{ Inhibition }} = \, \left( {{\text{Ac}} - {\text{As}}} \right) \, \times { 1}00/{\text{Ac}}$$

Here, Ac (absorbance of the control) and As (absorbance of the sample)^49,50^.

### Antimicrobial activity

#### Minimum inhibitory concentrations

Antimicrobial activity of different synthesized compounds was determined against *S. aureus*MTCC3160, *P. aeruginosa*MTCC1934, *E. coli*MTCC45, *P. mirabilis*MTCC3310*, C. albicans*MTCC183 and *A. niger*MTCC282 by using tube dilution method. For determining the antimicrobial potential dilutions of test and standard compounds were prepared in nutrient broth I.P. (bacteria) and sabouraud dextrose broth I.P. (fungi). After the incubation period the sterilized 0.9% NaCl solution was used to harvest the bacterial and fungal cultures from agar slant through proper shaking and then the suspensions of microorganisms were diluted with the sterile 0.9% NaCl solution to CFU count was adjusted by adjusting the density of microorganism suspension to that of 0.5 McFarland standards by adding distilled water. The number of CFU was determined by dilution pour-plate method. A serial dilution of 50 µg/mL, 25 µg/mL, 12.5 µg/mL, 6.25 µg/mL, 3.12 µg/mL and 1.56 µg/mL was used for determination of MIC. The samples tubes were incubated at 37 °C for 24 h (bacteria), at 25 °C for 7 days (*A. niger*), and at 37 °C for 48 h (*C. albicans*), and the results were recorded in pMIC^51–53^.

### Preservative effectiveness

White lotion USP was used for the evaluation of preservative efficacy of naringin derivatives. Selected derivatives of naringin were used as preservatives in amount equivalent to the standard taken in White lotion USP prepared as per the method of Khatkar et al*.* The synthesized compounds **1**–**8** in equimolar amount (0.0013 mol of methyl paraben) were used as novel preservatives by replacing standard preservatives sodium benzoate, methyl paraben and propyl paraben in both the preparations^13^.

### Challenge microorganisms

Standard microbial strains of *S. aureus* MTCC 3160, *P. aeruginosa* MTCC 1934, *E. coli* MTCC 45, *C. albicans* MTCC 183 and *A. niger* MTCC 282 were used as common contaminants as per USP criteria for preservative efficacy testing in the pharmaceutical preparations.

### Preparation of inoculums

The slants of *E. coli*, *P. aeruginosa,* and *S. aureus* were incubated at the 37 °C for 24 h. The slants of *C. albicans* were incubated at 37 °C for 48 h, whereas; the slants of *A. niger* were incubated at 25 °C for 7 days^54^.

### Test procedure

White lotions USP in final containers was used in the challenge test for preservative efficacy. The preparation was inoculated with a 0.5–1% volume of microbial inoculum having a concentration of 1 × 10^5^–1 × 10^6^ CFU/mL. Inoculated samples were made homogeneous after adding microorganism and incubated. The CFU/mL of White lotion USP was determined at interval of 0 days, 7 days, 14 days, 21 days, and 28 days in agar plates. Log CFU/mL of white lotion USP was calculated as not less than 2.0 log reductions from initial count on 14th day of incubation and no increase in CFU from 14th day count to 28th day in case of bacteria and no increase from the initial calculated count on 14th day and 28th day in case of fungi^55,56^.

### Stability studies of the selected preservatives

From the results of preservative efficacy study, selected compound was further evaluated for its stability as per the protocol provided in ICH guidelines. The selected compound was added in the final containers containing White Lotion USP. The preparation having standard preservative and test compound was stored at 40 ± 2 °C at 75% RH ± 5% RH (as per ICH guidelines) and was analyzed for the change in pH and cfu/ml at the time interval of 0, 1, 2, 3, 4, 5 and 6 months.

### Biological evaluation of selected preservatives

Finally, the selected most active compounds from preservative efficacy and stability study were evaluated for in vitro skin permeation, skin and eye irritation study. Naringin compound **7** was selected for their biological evaluation as per the following procedures:

### In vitro skin permeation study using Franz diffusion cell

The skin permeation study of selected derivative through transdermal was evaluated by using modified Franz diffusion cell apparatus with a glass diffusion cell along with a receptor and donor cell. The receptor cell has an internal volume of 10 ml and side arm allowed sampling of receptor fluid. The donor cell was clamped on to the top of the receptor cell. Water at 37 °C was circulated through the water jacket surrounding the receptor cell. Strat-M (a synthetic human skin) membrane was used for transdermal diffusion testing. Membrane was kept in freshly prepared solution of phosphate buffer (pH 7.4) and mounted between donor and receptor cell. The receptor was filled with phosphate buffer solution. A volume of 1 ml selected compound 0.1% solution was placed in donor cell, while the receptor cell fluid was kept under stirring and permeation for 120 min. At appropriate time, 1 ml sample was withdrawn from receptor cell at 5, 10, 15, 30, 60 and 120 min., replacing the withdrawn sample with fresh buffer solution. Samples were analyzed by measuring the absorbance at 291 nm. Concentration of different compounds at different time interval was calculated by standard plot^57,58^.

### Skin irritation test in rabbits

Draize test has based on the principle of skin damage caused by direct toxic action of irritant substances. The Draize 24-h patch test in rabbits has been utilized as the most widely used animal test for testing of primary irritant substances^59^. Test substance (0.5 g) was smoothly applied over the previously shaved rabbit’s skin on 6 cm^2^ area and covered with gauze patch. If, no irritation has been observed in initial test, then confirmatory test will be conducted in another two rabbits, patch was removed after 4 h of contact and observations were made after 1, 24, 48 and 72 h after patch removal. The dermal irritation scores were recorded on the basis of type and severity of lesions, and graded as per standard Draize test scoring criteria^60^.

### Eye irritation test rabbits

Test substance (100 mg/0.1 ml) was smoothly applied in rabbit’s eyes. No irritation was observed in initial test, (after the 1 h of administration), then confirmatory test was conducted and observations were noted. The grades of ocular reaction (conjunctivae, cornea and iris) will be observed as per OECD guidelines and recorded at an interval of 1, 24, 48, and 72 h after the application of test substance. Different grades for eye irritation severity were recorded as per standard criteria.

### Statistical analyses

All the data was represented as mean ± standard deviation (SD) for three triplicates of each sample. One-way ANOVA test at a significance level of 0.05 (p < 0.05) using MS excel statistical tool was used to analyze the experimental data.

### Ethics approval and consent to participate

Institutional Animal Ethical Committee of M.D. University, Rohtak, India has approved the experimental protocol for use of rabbits vide letter no. 1767/GO/Re/S/14/CPCSEA, dated- 31/08/2017. Authors also confirming that all experiments were performed in accordance with relevant guidelines and regulations.

## Results and discussion

### Molecular docking

On the basis of molecular docking and ADMET parameters of proposed naringin derivatives as compound **1** to **8** were selected for further synthesis and biological evaluation. Compound **6** and **7** exhibited dock score (− 7.98 and − 8.45, respectively) and binding energy (− 65.35 kJ/mol and − 69.22 kJ/mol, respectively) as compared to the dock scores (− 5.18, − 5.06, − 5.12) and binding energies (− 37.16 kJ/mol, − 25.41 kJ/mol and − 23.15 kJ/mol) of standard drugs ciprofloxacin, ampicillin, and fluconazole, respectively. Docking results of compound **7**, showed the formation of four hydrogen bonds between residues Ala 602, Val 399, Cys 300 and Thr 302 with hydroxyl and oxygen atom of synthesized ligands. Hydrophobic interactions were seen among residues Ser 303 and Glu 488. Compound **6** has been attached to Gln 348, Thr 302 and Val 602 with hydroxyl as well oxygen atom of synthesized compounds by four hydrogen bonds and was also found to interact hydrophobically with Glu 488 and Leu 601 residues. The results of molecular docking for different ligands within G-6-P synthase pocket and their interaction with different amino acid residues have been shown in Table 1. Here, the inhibition of G-6-P synthase enzyme further evaluated by the outcomes of the inhibition likes antimicrobial activity. This further made the clearance behind the inhibition of G-6-P synthase enzyme by different proposed molecules.

**Table 1:** Results of molecular docking for proposed naringin derivatives.

Chemical Structure of docked compounds	Results of docking		Interaction diagram	Bonding in enzyme pocket
	Docking score	Binding energy (kJ/mol)		
Compound **1**	− 6.61	− 61.17		H-bond: Glu 488, Lys 603Ser 349, Thr 352 Hydrophobic interactions: Val 399. Ala 400, Leu 601, Cys 300, Ala 300
Compound **2**	− 5.47	− 54.22		H-bond: Ala 602, Gly 329, Lys 603 Hydrophobic interactions: Cys 300, Leu 601, Val 606, Thy 332
Compound **3**	− 2.14	− 31.50		H-bond: Thr 302, Ala 602, Hydrophobic interactions: Thr 352, Glu 438
Compound **4**	− 5.30	− 47.65		H-bond: Thr302, Cys 300 Hydrophobic interactions: Glu 488, Ser 303
Compound **5**	− 3.39	− 39.79		H-bond: Thr302, Cys300, Ala 602 Hydrophobic interactions: Thr 352, Glu 488, Ser 303
Compound **6**	− 7.98	− 65.35		H-bond: Gln 348, Thr 302 and Val 602 Hydrophobic interactions: Glu 488, Leu601
Compound **7**	− 8.45	− 69.22		H-bond: Ala 602, Val 399, Cys 300 and Thr 302 Hydrophobic interactions: Ser303 andGlu 488
Compound **8**	− 6.55	− 53.23		H-bond: Ala 602, Val602, Thr 310 Hydrophobic interactions: Glu 488
Naringin	− 6.06	− 49.50		
**Standards**				
Streptomycin	− 5.79	− 34.30		
Ciprofloxacin	− 5.18	− 37.16		
Ampecillin	− 5.06	− 25.41		
Fluconazole	− 5.12	− 23.15		

### ADME study

Different ADMET parameters of proposed aesculin derivatives were determined in silico by QikProp application (Schrodinger LLC) and PreADMET software. The parameters were analyzed and were compared with the standard values**.** Here, QPPCaco descriptor determined the Caco-2 cell permeability and predicted value defined the barrier between gut and blood system. QPlogBB was the blood/brain partition coefficients determined to be used as a parameter contributing the entry of drugs to the central nervous system. QPPMDCK was used for the estimation of oral absorption. QPlogKp was the descriptor that determined the dermal penetration. The lower values logKp was considered to have low skin permeation and all the selected aesculin derivatives (marked yellow) were having the best suited values range and were to be selected as preservative for topical formulation. QPlogKhsa descriptor determined the binding of drugs to plasma proteins^61–65^. The different ADME parameters of proposed naringin derivatives have been represented in Table 2. All the results were expressed as mean ± standard deviation (n = 3) and results were found significant p < 0.05.

**Table 2:**
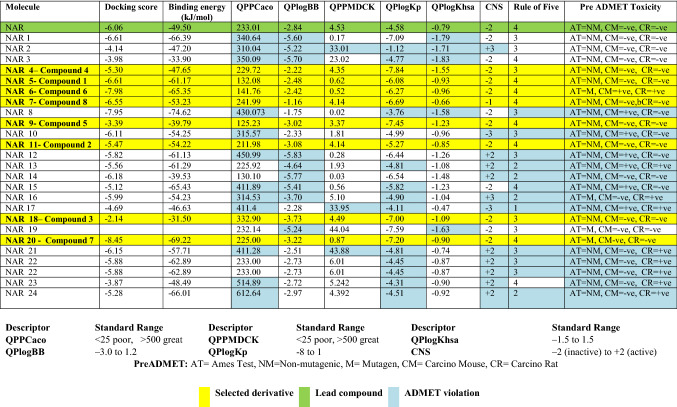
Results of molecular docking and ADMET parameters for proposed naringin derivatives.

QPPCaco: Caco-2 cell is a model for the gut-blood barrier; QPlogBB: Predicted brain/blood partition coefficient; QPPMDCK: MDCK cells are considered to be a good mimic for the blood brain barrier; QPlogKp: Predicted skin permeability; QPlogKhsa: Prediction of binding to human serum albumin; CNS: Predicted central nervous system activity.

### Chemistry

Scheme 1 was used for the synthesis of selected naringin derivatives. The FTIR data revealed the formation of compound **1** to **8**, which was confirmed by peak shifted from 1730 cm^−1^ (–C=0) to 1633 cm^−1^, − 1690 cm^−1^ (–C=N–) and appearance of 750 cm^−1^ (–Cl–) for compound 1, 1690 cm^−1^ (–C=N–) for compound 2, 1632 cm^−1^ (–C=N–) for compound 3, 1629 cm^−1^ (–C=N–) for compound 4, 1632 cm^−1^ (–C=N–) for compound 5, 1633 cm^−1^ (–C=N–) for compound **6**, 1690 cm^−1^ (–C=N–), for compound **7** and 1693 cm^−1^ (–C=N–) for compound **8** respectively. The change in chemical shift value, coupling constant and multiplicities were analyzed by 1HNMR and 13C NMR signals of synthesized compounds. The FTIR, 1H NMR, 13C NMR data, mass spectroscopy, and elemental analysis confirmed the chemical structures of synthesized naringin derivatives.

### Antioxidant activity

#### DPPH radical scavenging activity

DPPH free radical scavenging assay confirmed that compounds **7**, **6** and** 1** possessed good antioxidant potential with IC_50_ 6.23 ± 0.03 µM, 7.03 ± 0.03 µM, 7.31 ± 0.06 µM, respectively as compared to standard l-ascorbic acid IC_50_8.11 ± 0.06 µM. Antioxidant potential (IC_50_) of other synthesized naringin derivatives Compound 2, 3, 4, 5, 8 and naringin itself has been found as 14.52 ± 0.40 µM, 10.62 ± 0.01 µM, 18.77 ± 0.06 µM, 11.32 ± 0.16 µM, 20.9 ± 0.26 µM and 6.36 ± 0.36 µM, respectively. Here, the better antioxidant property of amygdalin derivative shall be useful in the preservation of food, cosmetics, and pharmaceuticals^66^.

### Antimicrobial activity

#### Minimum inhibitory concentrations

The recorded pMIC values, revealed compound **7** as most effective antimicrobial (pMIC 2.07, 2.37, 2.07, 2.37, 1.77, and 1.77 µM/mL for *P. mirabilis, P. aeruginosa, S. aureus*, *E. coli, C. albicans, *and *A. niger, *respectively), as compared to the standard drugs ciprofloxacin (pMIC 1.12, 1.42, 1.12, and 1.42 µM/mL for *P. mirabilis, P. aeruginosa, S. aureus, *and *E. coli, *respectively), Ampicillin (pMIC 1.14, 0.84, 0.84, and 1.74 µM/mL for *P. mirabilis, P. aeruginosa, S. aureus, *and *E. coli, *respectively) and fluconazole (pMIC 1.08, and 1.38 µM/mL for *C. albicans,* and *A. niger, *respectively). The results of antimicrobial activity revealed that the synthesized compounds have antimicrobial potential as compared to standard drugs as shown in Table 3. The probable mechanism of antimicrobial activity of naringin derivatives may be due to the better inhibition of G-6-Psynthase.

**Table 3:** The pMIC values (µM/mL) of synthesized naringin derivatives against different standard microbial strains.

Compound(s)	pMIC values in µM/mL					
	*P. mirabilis*	*P. aeruginosa*	*S. aureus*	*E. coli*	*C. albicans*	*A. niger*
Compound **1**	1.44	1.74	1.44	1.14	1.44	1.44
Compound **2**	1.12	1.42	1.12	1.42	1.12	1.42
Compound **3**	< 1.08	1.38	1.38	1.08	< 1.08	< 1.08
Compound **4**	1.13	1.13	1.43	1.13	< 1.13	< 1.13
Compound **5**	1.11	1.11	1.41	1.11	< 1.11	< 1.11
Compound **6**	2.35	2.35	1.75	1.75	2.35	2.35
Compound **7**	2.07	2.37	2.07	2.37	1.77	1.77
Compound **8**	1.13	1.13	1.43	1.43	1.13	1.43
Naringin	< 1.06	< 1.06	< 1.06	1.06	< 1.06	< 1.06
Ciprofloxacin	1.12	1.42	1.12	1.42	–	–
Ampicillin	1.14	0.84	0.84	1.74	–	–
Fluconazole	–	–	–	–	1.08	1.38

### Preservative efficacy study

**Figure 1:**
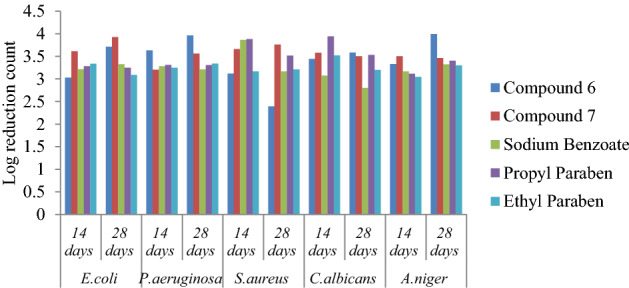
Preservative efficacy of compound **7** in White lotion USP and the degree of microbial log reduction.

The highly active antimicrobial compounds **6** and **7** in the series were selected for the evaluation of preservative efficacy. The results of preservative efficacy testing were performed in triplicate and have been reported as mean values in Table 4. Compound **7** showed the values of log CFU/mL reduction within the prescribed limit and the results were comparable to that of the standard preservatives sodium benzoate, propyl paraben and methyl paraben. Result of compound 6 showed a less than 2.0 log reductions from initial count on 14 days and number of CFU/ml values of preservative efficacy against some microbial strains increased on the 14th day to 28th day as compare to standard preservatives sodium benzoate, propyl paraben and methyl Paraben and found significant with p < 0.05.Preservative efficacy of compound **7** in White lotion USP and degree of microbial log reduction have been represented in Fig. 1.

**Table 4:** Log CFU/ml values of the selected compound **7** and **6** in White lotion USP.

Compounds	*E. coli*		*P. aeruginosa*		*S. aureus*		*C. albicans*		*A. niger*	
Cfu/mL after days	14 days	28 days	14 days	28 days	14 days	28 days	14 days	28 days	14 days	28 days
Compound **6**	3.21 ± 0.010^a^	3.92 ± 0.12^b^	2.20 ± 0.10^c^	3.56 ± 0.05^d^	3.66 ± 0.01^e^	3.76 ± 0.02f.	3.58 ± 0.13^ g^	3.50 ± 0.02^ h^	3.50 ± 0.02^i^	2.46 ± 0.03^j^
Compound **7**	2.03 ± 0.03^a^	2.71 ± 0.02^b^	2.63 ± 0.02^c^	2.96 ± 0.03^d^	2.11 ± 0.01^e^	2.39 ± 0.04f.	2.44 ± 0.08^ g^	2.58 ± 0.08^ h^	2.33 ± 0.04^i^	2.99 ± 0.01^j^
Sodium benzoate	2.23 ± 0.01^a^	2.33 ± 0.24^b^	2.22 ± 0.16^c^	2.21 ± 0.03^d^	2.53 ± 0.04^e^	2.16 ± 0.04f.	2.07 ± 0.08^ g^	2.80 ± 0.08^ h^	2.16 ± 0.01^i^	2.32 ± 0.01^j^
Propyl paraben	2.20 ± 0.57^a^	2.24 ± 0.36^b^	2.31 ± 0.01^c^	2.30 ± 0.01^d^	2.73 ± 0.02^e^	2.56 ± 0.01f.	2.90 ± 0.02^ g^	2.53 ± 0.01^ h^	2.13 ± 0.06^i^	2.43 ± 0.01^j^
Ethyl paraben	2.36 ± 0.02^a^	2.00 ± 0.14^b^	2.24 ± 0.36^c^	2.34 ± 0.01^d^	2.16 ± 0.04^e^	2.10 ± 0.02f.	2.50 ± 0.01^ g^	2.20 ± 0.01^ h^	2.03 ± 0.04^i^	2.30 ± 0.08^j^

Initial concentration of microbes in inoculum 1 × 10^5^–1 × 10^6^.

CFU = Colony forming unit, all experiments were conducted in triplicate (n = 3) and the mean values are presented. Different letters mean p < 0.05 in each line by One-way ANOVA test.

### Stability study

Results of stability study revealed that the pH of White lotion USP samples were in range of 5.5–6.0.The results of the microbial study indicated that no microbial growth was observed in samples containing compound **7** over a period of six months period as per ICH guidelines. These results indicated that the product was stable as compared to standard preservative with added naringin compound **7** as preservative. The results of stability study were performed in triplicate and were reported as mean values. Results for microbial growth and pH changes also found to be significant at p < 0.05.

### Biological evaluation of selected preservatives

#### In vitro skin permeation study using Franz diffusion cell

In vitro skin permeation study of selected compound **7** was performed by using Franz diffusion cell having receptor and donor cell. Concentration of compound **7** at different time interval was calculated by standard plot. It was observed from the skin permeation data with different time intervals that maximum amount of drug was released within 120 min. It was concluded from the skin permeation data as shown in Table 5 that the maximum amount of drug released within first 120 min i.e**.** 45.05%. The total amount of selected compound **7** that was permeated through selected skin graft was found to be 4.27 mg (0.0013 mol of sample taken).

**Table 5:** Percentage of compound **7** diffuse through Franz diffusion cell.

Time (min)	Absorbance	Compound **7** released (μg)	% Released	% Unreleased
0	0	0	0	100
5	0.22	15.14	15.14	84.85
10	0.34	23.92	23.92	76.07
15	0.53	37.35	37.35	62.64
30	0.57	40.17	40.17	59.83
60	0.60	42.5	42.5	57.5
120	0.64	45.07	45.07	54.93

#### Skin irritation test of naringin derivative in rabbits

**Figure 2:**
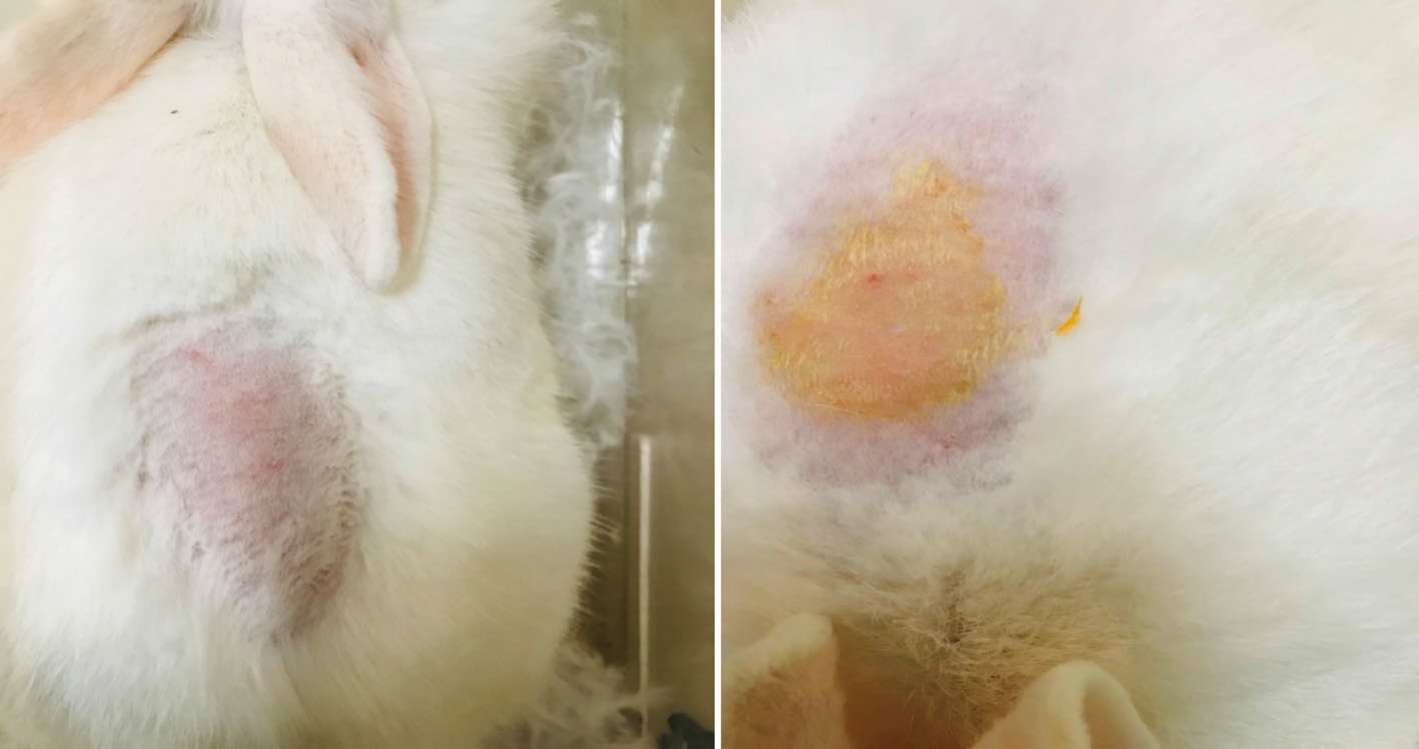
Skin erythema before and after application of test compound.

The dermal irritation scores were evaluated by the type and severity of the lesions produced. All the rabbits were checked for the reversibility of any skin reaction up to 14 days, by evaluating skin irritation responses i.e. alopecia, hyperplasia, hyperkeratosis and scaling. Scoring of the dermal reaction was done with the standard OECD guideline-404**.** The test compound **7** did not showed any type of dermal reaction and was considered as non-irritant. No skin erythema and edema formation was observed during test. The irritation score for erythema and edema in all the rabbits were found have Score 0. Skin area of rabbit used for testing of compound **7** before and after application have been shown in Fig. 2.

#### Eye irritation test of naringin derivative in rabbits

**Figure3:**
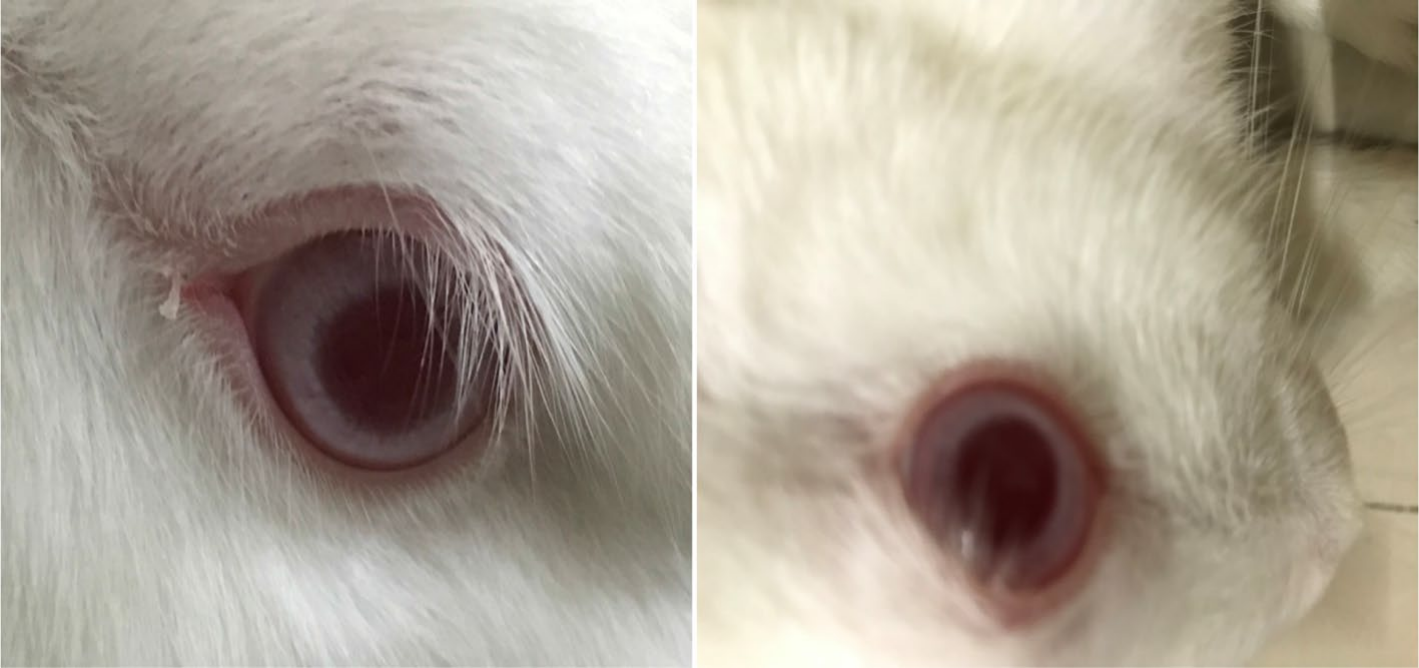
Before and after instillation of test compound.

Compound **7** was also evaluated for eye irritation test in rabbits. The observations were checked for reversibility of any eye irritation reaction up to 21 days of test compound application. The grades of ocular toxicity reactions were observed and recorded at 1, 24, 48, and 72 h following test substance application as per standard OECD guideline-405. The test compound compound **7** did not showed any type of eye irritation reaction and was considered as non irritant for eyes. No ulceration, conjunctivae redness and chemosis were observed in eye irritation test. The irritation score for cornea chemosis, conjunctivae and iris in all the rabbits were found to have 0. Changes in produced in the eyes of the rabbits used for testing of compound **7** before and after application have been shown in Fig. 3.

#### Structure activity relationship (SAR) studies

**Figure 4:**
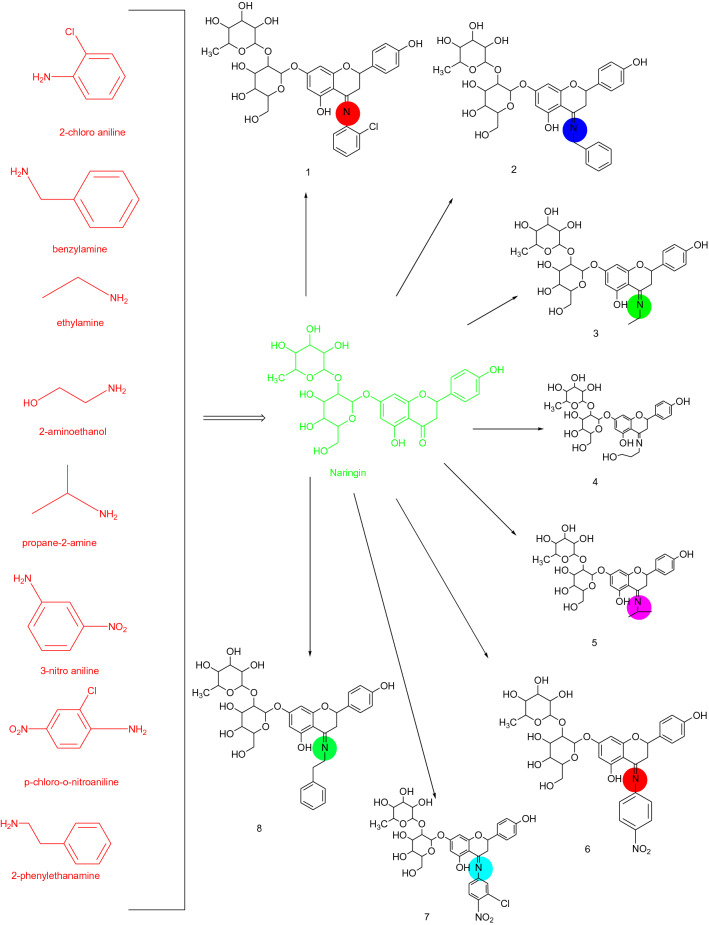
Design of the strategy of naringin derivatives for G-6-P synthase inhibition and antioxidant activity.

Design approach of naringin derivative for G-6-P inhibition and antioxidant activity has been represented in Fig. 4. The structure activity relationship of the synthesized naringin derivatives with their antioxidant activity results have been summarized as:

1. The substitution of carbonyl group of naringin with aliphatic aliphatic amines decreased the biological activity i.e. compounds **3**, **4**, **5** shows lower activity as compared to compounds **1**, **2** and **7**.
2. The substitution of carbonyl group of naringin with aromatic amide ring attached directely to Naringin enhanced the biological activity.
3. The presence of electronegative halides on ortho position of aromatic amide ringj enhanced the biological activity (compound **1** and **7**).
4. The presence of nitro group on para position of aromatic amide ringj enhanced the biological activity (compound **6** and **7**).

## Conclusion

The above mentioned wet and dry laboratory study results clarify the mechanism of enzyme G-6-Psynthase inhibition. The most active compound **7** i.e. 2-(4,5-dihydroxy-2-(5-hydroxy-2-(4-hydroxyphenyl)-4-(4-nitrophenyl amino)-3,4-dihydro-2*H*-chromen-7-yloxy)-6-(hydroxymethyl)-tetrahydro-2*H*-pyran-3-yloxy)-6-methyl-tetrahydro-2*H*-pyran-3,4,5-triolshowed antioxidant, antimicrobial, better preservative efficacy and prevent the change in pH as well microbial count of formulation for food as well as pharmaceutical products, which were in agreement with the results of molecular docking and highlight the mechanism of their preservative activity. Therefore, the synthesized amygdalin derivatives can be used as novel food and pharmaceutical preservatives to prevent them from microbial degradation.

## Data Availability

The datasets used and/or analyzed during the current study are available from the corresponding author on reasonable request.

## Abbreviations

ADMET: Absorption, distribution, metabolism, excretion and toxicity
G-6-Psynthase: Glucosamine-6-phosphate synthase
DPPH: 2,2-Diphenyl-1-picrylhydrazyl
UDP-N-acetyl glucosamine: Uridine diphosphate *N*-acetylglucosamine
FTIR: Fourier-transform infrared spectroscopy
^1^H NMR: Proton nuclear magnetic resonance
^13^C NMR: Carbon 13 nuclear magnetic resonance
UV: Ultra violet
TLC: Thin layer chromatography
IC_50_: Inhibitory concentration
MIC: Minimum inhibitory concentrations
CFU: Colony forming unit
HBA: Hydrogen bond acceptor
HBD: Hydrogen bond donor
MW: Molecular weight
MTCC: Microbial type culture collection
DMSO: Dimethyl sulfoxide
BOD: Biological oxygen demand
USP: Unites state pharmacopoeia
PDB ID: Protein data bank identification
OPLS: Optimized potential for liquid simulations
